# QRBT: Quantum Driven Reinforcement Learning for Scalable Blockchain Transaction Processing

**DOI:** 10.1371/journal.pone.0342689

**Published:** 2026-02-19

**Authors:** Kranthi Kumar Lella, Shiva Rama Krishna Mallu

**Affiliations:** 1 Manipal Institute of Technology, Manipal Academy of Higher Education, Manipal, India; 2 Faculty of Operations & IT, IBS Hyderabad, The ICFAI Foundation for Higher Education, Hyderabad, Telangana, India; Maulana Abul Kalam Azad University of Technology West Bengal, INDIA

## Abstract

Scaling and Latency Issues Processing blockchain transactions is a challenge with respect to throughput and latency, while also being resilient against quantum adversaries. To overcome these,m this research presents QRBT:Quantum Driven Reinforcement Learning for Scalable Blockchain Transaction Processing, a Quantum Based Reinforcement Learning Framework, where variational quantum circuits and QKD are utilized jointly with an actor-critic paradigm of RL to improve consensus strategies and transaction validation. The system adopts a four tier architecture quantum computation layer, reinforcement learning layer, blockchain security layer and transaction processing layer to support adaptive policy optimization driven by quantum enhanced state encoding and circuit refinement. Our experimental results with Ethereum mainnet traces and synthetic workloads further show significant improvements in system performance the transaction latency is reduced by 91.264% at level-1, and 76.298% at level-5. The cryptographic security under quantum attack approximation remains the strongest 96.152% at Level-1, 83.728% at Level-5, scalability enhanced 92.635% throughput improvement of Level-1, 79.512 contested TPS of the block stage within each mining epoch, consensus energy consumption becomes the least among all systems 69.957kWh at Level-1, 84.963kWh of computational cost at level-5. Cost for reinforcement learning convergence stabilizes quite efficiently, further lifting from 81.937 percent at Level-1 to 92.746 percent at Level-5 and showing the superiority over other QAOA, QAOA-RL, QSVT,QPSO and AQO baselines. The results show that QRBTmaintains the high throughput, security strength and energy efficient consensus simultaneously in their resistance to quantum attacks. The results show that quantum assisted reinforcement learning is a scalable and secure approach for the next generation of blockchain systems.

## 1 Introduction

The rapid evolution of blockchain technology has fundamentally transformed digital transaction processing, establishing decentralized networks that enable secure, transparent, and immutable record-keeping across various industries. However, contemporary blockchain systems face significant challenges in achieving optimal performance while maintaining robust security guarantees, particularly as transaction volumes scale exponentially and quantum computing threats emerge on the horizon. Traditional consensus mechanisms and cryptographic protocols, while effective in classical computing environments, demonstrate inherent limitations in processing efficiency, energy consumption, and vulnerability to quantum attacks. The blockchain trilemma, balancing security, scalability, and decentralization, remains a persistent challenge that requires innovative approaches combining cutting edge technologies to address the computational complexity and security requirements of next generation distributed systems. Blockchain technology has become the foundational networking layer for performing secure and decentralized digital exchange, allowing transparent auditability and trustless coordination within application domains spanning verticals such as the finance, healthcare, supply chain, and IoT ecosystems. However as the transactions increases and network participation increases, existing blockchain envisions severe scalability challenges including high latency, throughput bottleneck and energy consumption of consensus protocol. Current solutions, from the layer-2 scaling on-chain to better consensus mechanisms can bring more equilibrium to the trilemma of blockchain between security, scalability and decentralization but can not truly balance it in a trusted way.

In order to overcome these challenges, we propose QRBT: Quantum Driven Reinforcement Learning for Scalable Blockchain Transaction Processing, a hybrid methodology that combines quantum computing and deep reinforcement learning for overall optimization of the blockchain. QRBT integrates VQC-based quantum state encoding, a multi actor quantum RL architecture, and a post quantum security layer using PQC and QKD to enable adaptive decision making. By jointly optimizing transaction validation, latency, throughput scaling, and energy efficient consensus, the model achieves real-time resilience under diverse workloads and adversarial conditions. Extensive simulations on Ethereum mainnet traces and stress models show that QRBT enhances quantum security without degrading latency or throughput. Overall, the results highlight QRBT as a scalable and solution for secure blockchain transaction processing.

Research outlines significant advancements in secure, scalable, and quantum resilient blockchain transaction processing through several key contributions:

- **Hybrid QRBT Framework**: A framework combining quantum computing and reinforcement learning for optimized transaction validation and consensus.
- **Quantum-Resistant Security Architecture**: Integration of post-quantum cryptography and quantum key distribution to enhance resilience against quantum attacks.
- **Adaptive Optimization and Scalability**: Reinforcement learning-driven optimization for reduced latency and improved throughput.
- **Comprehensive Performance Evaluation**: Rigorous simulations benchmarking QRBT against quantum and classical algorithms across key performance metrics such as Transaction Processing Latency Reduction, Cryptographic Security, Blockchain Scalability, Energy Efficient Consensus Quantum, RL Convergence Rate.
- **Practical and Theoretical Advancements**: A four-layer architecture with mathematical models unifying quantum and reinforcement learning for blockchain security and efficiency.

## 2 Related work

Blockchain QRL in the context of e-mobility application improves the peer-to-peer energy trading at local level within e-mobility micro-grid by considering double auctions, consortium blockchain consensus mechanism and Grover based quantum parallelism for dynamic pricing and scheduling [1]. Faster convergence, greater social welfare and cheaper market prices are observed in the experiments on Rigetti QVM with 16 microgrids and 60 EVs vs. Hyperledger Fabric consumes less energy compared to traditional blockchain-based topologies [2]. A systematic IoT security survey surveys blockchain, ML, cryptosystems and quantum computing to defend IoT deployments benchmarking computational efficiency, detection accuracy and quantum resilience using OMNET++, MATLAB,AWS, Ethereum deployment [3]. QRL optimization through VQC reduces the number of trainable parameters in Frozen Lake, Cart Pole, and Lunar Lander while keeping competitive reward performance under PennyLane and IBM QASM imulations Hybrid quantum classical approaches exhibit improved convergence with respect to classical RL and competitive performance for extended training and in presence of quantum noise, showing great promise when dealing with complex environments [4]. PQ Kyber512 with ZK-Proof PQSecure Cryptocurrency wallets constructed using post-quantum signatures, secure biometric authentication will be integrated in the future For POST-QUANTUM CRYPTOGRAPHY KYBER suits best to following where public and private keys are not to exceed 1000 bytes [5].

The challenge of resource efficient Metaverse construction using unmanned aerial vehicles has been addressed through the development of quantum collective reinforcement learning [6]. This approach leverages quantum parallelism to accelerate training and employs collective learning to enhance adaptability in dynamic environments, thereby optimizing task offloading. Key performance indicators include reward maximization, offloading rates, training speed, and generalization capability. Comparative simulations against classical algorithms Proximal Policy Optimization,Soft Actor-Critic demonstrate that QCRL achieves superior convergence and resource utilization. Notably, QCRL reduces latency, improves scalability, and enables rapid UAV adaptation, with future research aimed at addressing emerging security concerns [7]. To address quantum computing threats and cross-chain security challenges, the BSCTQCAT framework introduces a blockchain smart contract architecture designed for quantum resistance and secure interoperability [8]. Framework employs lattice based digital signatures and a reputation driven identity agent layer to secure cross chain interactions, achieving improved quantum resistance and interoperability on HyperLedger Fabric. Additionally, a quantum actor–critic system optimizes spatio temporal resource allocation on NISQ devices, demonstrating superior scalability and real time performance over classical RL and random walk baselines. For dynamic resource allocation,task offloading in IoT systems utilizing mobile edge computing, a quantum empowered deep reinforcement learning framework has been introduced [9]. This approach exploits quantum superposition, entanglement, and a modified Grover algorithm to accelerate the exploration-exploitation trade-off, thereby reducing computational complexity and enhancing learning efficiency. Simulations in dynamic IoT environments demonstrate that QDRL surpasses classical deep reinforcement learning and other benchmarks in energy efficiency and convergence speed. Future work will extend this framework to digital twin networks and hybrid connected vehicular systems.Finally, a quantum blockchain driven Web 3.0 framework has been proposed to overcome the vulnerabilities of classical blockchain systems [10].

The Quantum Entropy Q-Learning model combines superposition, entanglement and entropy-based anomaly detection to improve smart grids DDoS detection and it is better than the classic models and the entropy Q-Learning in terms of precision, recall, F1-score with convergence index on the CICIDS-2019 dataset using MATLAB Python - Qiskit [11]. In finance and blockchain, QAE, Grover search and VQAs underpin expedited pricing of derivatives, risk models and consensus to deliver power efficient optimization on NISQ-era machines despite limitations [12]. Quantum assisted blockchains and QKD-enabled protocols make CIoT infrastructure resilient to such attacks, minimizing the attack success rate and verification time while enhancing scalability over Cirq and IBM Qiskit simulators [13]. A novel PPO–LSTM–CNN hybrid model incorporating sentiment analytics outperforms other models in predicting cryptocurrency for BTC, ETH, and LTC, and exhibits higher Sharpe, Sortino ratios along with cumulative return when using Stable Baselines3 [14] to trade on gym anytrading. Hybrid QRL methods involving variational quantum circuits with DQN and A2C achieve reduction of trainable parameters, or even more, while achieving competitive rewards inFrozen Lake, Cart Pole and Lunar Lander. Despite the difficulty in dealing with quantum noise and a long way of training, QRL has shown reasonable space efficiency, faster convergence rate and superior robustness for challenging RL problems as we considered [15].

Empirically [16] introduces the joint utilization of machine learning and quantum computing for improved IoT security, combining SVMs, neural networks, QKD and quantum neural networks in efforts to reinforce anomaly detection and encryption, with evaluations carried out in terms of accuracy, computation speed and resistance to quantum-attacks that put forth such concepts both into light as well as into hardware bounds. Additionally [17] presents MRL-PoS, a multi agent reinforcement learning proof-of-stake mechanism designed to detect and punish defrauding validators with reputation scores in order to enhance fairness and security on four adversarial scenarios but still requires validation at large scale using real world environment. Furthermore [18] presents a healthcare architecture using FL, BC and QC for high throughput, low latency and immutability, privacy security with reference to personalized medicine system. Moreover [19] combines blockchain to double dueling Q-network DRL to maximize IoT task offloading, and the computational costs system cost by and energy consumption by, via simulations and Ethereum verification. Inaddition [20] suggests a quantum inspired blockchain for Internet of things in smart cities with a quantum walk based hash functions which is evaluated by NIST SP 800-22 randomness tests and its sensitivityanalysis to defend from tampering the man-in-middle attack. The theoretical implementation in smart water utilities validates secure authentication and encrypted data transfer over quantum-secure channels, thus effectively ensuring the security of IoT communication.

Subsequently [21] proposes a dynamic cryptocurrency portfolio optimization with deep reinforcement learning and Monte Carlo Tree Search that achieves cumulative return,, of the maximum Sharpe ratio, controlled draw down in extreme markets while outperforming mean variance based baselines. QBC-ZKPAF framework is proposed in [22], Combining Zero-Trust Architecture, Post-Quantum Cryptography, and Blockchain by Reinforcement-Lattice KeyGen DQN based Adaptive Selection with Privacy Preserving Authentication through ZKP. It achieves privacy preservation, TPS, Joules energy consumption and access control with high scalability and robustness against MQTs on Edge-IIoTset [22]. Reinforcement Learning Based Deep FEFM model leverages the deep feature fusion, RL-driven policy fine tuning and non linear optimization to adaptively adjust blockchain consensus with lower latency and energy consumption and better scalability. We’re so far doing amazing for a white pager there but it’s actually performing well on simulated networks with, latency, energy consumption and fault tolerance beating PoW/PoS [23]. Explores the strength of block-chain when it comes to cryptographic vulnerabilities from quantum threats and compares RSA-2048/ECC-256 against Post Quantum Cryptosystems like Kyber-1024 and SPHINCS+ by showing reduced decryption immunity at larger qubit size but higher resistance with PQC despite extra computational cost [24]. A privacy friendly function and a Reference architecture, relying on Reinforcement Learning and HE secure computation enabling adaptive decision making, with quantum safe key exchange and digital signature for strong cloud-edge security. Simulations demonstrate that our proposed method can reduce the communication amount, increase delivery ratio, and operate more securely compared to existing methods [25].

Consequently [26] investigates quantum threats to blockchain, bearing out that RSA and ECC are severely threatened while hash based, lattice based cryptography and QKD can achieve long term security, revealing that the proactive use of quantum resistant algorithms is imperative for secure post quantum blockchain systems. On the ESP32 microcontroller, a hardware accelerated version of AQCU (Dilithium-5) has been proposed [27], which achieves key generation time, signing time and verification time on average using PQClean libraries. we use these figures to demonstrate that practical post quantum security is feasible for low cost IoT healthcare systems. A Blockchain-Based Deep Reinforcement Learning for IIoT healthcare is proposed in by embedding multi agent DRL with PoW and optimizing task scheduling, leading to makespan reduction and security improvement on EdgeXFoundry than DQN and DDPG, even if higher energy consumption but limited scalability. Hence [28] combines quantum cryptography and blockchain towards CBDCs and misinformation reduction, adopting QKD as well as quantum safe algorithms to enhance transparency, the integrity of transaction and decentralized verification in election scenarios. Hybrid quantum blockchain infrastructure of Web 3.0 that uses QKD, teleportation and post quantum cryptography to protect against new threats, increase scalability, throughput and privacy protection in decentralized system [29]. Use case scenarios from smart cities and health care prove WiBUD’s potential to increase resilience and security in next generation Web 3.0 environments [30].

Reinforcement learning blockchain framework for EV charging, in which Deep Q-Networks learn to make charging decisions with the knowledge of state-of-charge, dynamic pricing and trading locations, while a Proof of MCS protocol rewards mobile charging stations that help distant EVs for cost and reliability better than existing infrastructure. In simulations on OpenAI Gym and using real-world data companies, energy availability ratios, higher stakeholder payoffs and fraud free transactions are achieved by deploying the Avalanche blockchain infrastructure allowing to scale-up e-mobility services [31]. A quantum smart grid adversarial black-box attack analysis integrated with a quantum voting ensemble (SVM–KNN–NB) based cybersecurity system for smart grid [32] that can detect attacks, while maintaining low latency blockchain throughput is introduced. AI cryptography convergence for blockchain financial systems is investigated in [33] making use of predictive analytics, anomaly detection, and RL-powered consensus to optimize key management, security robustness and transaction performance. Therefore [34] includes reinforcement learning in SOAR environments with Q-Learning, DQN, and PPO to achieve adaptive threat response and ongoing optimization by simulating cyber attacks and case studies. Consideration shows that the detection accuracy is higher, the response time is shorter, and the resource allocation becomes better, which reflects RL’s role in resilient automated cybersecurity.

Quantum computing is integrated with advanced cryptographic schemes to overcome the real-time security limitations of classical methods, using quantum superposition, entanglement alongside algorithms such as Shor’s and Grover’s. Secure frameworks are reinforced with Quantum Key Distribution for robust key exchange, with effectiveness evaluated using metrics like Quantum Bit Error Rate and key generation rates, demonstrating enhanced security against both classical and quantum threats, though challenges such as qubit decoherence, system integration persist [35]. The transformative potential of quantum computing in the combat of financial crimes is highlighted through the use of quantum machine learning and AI techniques including quantum SVMs, quantum neural networks, quantum clustering, which enable the analysis of large datasets and the detection of complex patterns, with performance assessed by accuracy, processing speed, detection rates, scalability [36]. Furthermore, a novel framework for secure and efficient data transmission in smart grid Advanced Metering Infrastructure systems integrates QKD with multi-objective reinforcement learning, where a Proximal Policy Optimization agent optimizes routing based on energy, latency, reliability,security, achieving 750 J energy use, 55 ms latency, and 96% security in NS-2 simulations [37] Reinforcement learning and demand response techniques are applied to optimize peer-to-peer energy trading systems, enhancing decentralized market efficiency and resilience. Integration of advanced machine learning, battery storage, and blockchain technologies is a significant focus of analysis. Multi-agent and deep reinforcement learning approaches are evaluated for dynamic energy transaction management. Demand response strategies are explored for real-time supply-demand balancing in energy markets.Performance metrics highlight reductions in electricity costs, improved trading efficiency, and peak demand reduction [38]. Blockchain and IoT integration address security and data integrity, countering physical, software, and network vulnerabilities.A conceptual framework combining game theory, machine learning, and cyber deception is proposed for security optimization, measuring privacy, integrity, authentication, and attack resistance [39]. Quantum computing convergence is examined using ABCD analysis, assessing benefits, hardware progress, error correction, and algorithmic advancements across industries [40].

Furthermore [41] combines quantum key distribution and blockchain with tensor network based quantum deep reinforcement learning to optimize resource scheduling in vehicular edge computing, and can get faster convergence and higher efficiency. [42]. The authors advance a federated learning and quantum inspired DRL framework considering lattice based cryptographic hashing under blockchain for decentralized anomaly detection and quantum resistant security aspects to food supply chain. Subsequently [43] Quantum cryptography for blockchain security by combining it with federated deep reinforcement learning based intrusion detection to ensure privacy, robustness and decentralized threat mitigation under distributed IoT nfrastructure. Federated RL with adaption among blockchain nodes enhances real-time intruder response, and deep RL combined with Bayesian trust inspection ensures trusted shared vehicular data systems. [44]. A large model framework for vehicular edge networks optimizes task offloading and resource management with quantum computing, DRL, and LSTM networks [45]. IoT environments face significant security concerns; current solutions span blockchain, machine learning, cryptography, and quantum computing, with ongoing reviews comparing these mechanisms [46]. A taxonomy of these approaches is presented, highlighting their respective strengths, limitations, and suitability for various security requirements. The study discusses the benefits and challenges of each mechanism, offering insights for future secure IoT deployments.

## 3 Proposed system architecture

Sect 3 presents a detailed discussion of the proposed system QRBT: Quantum-Driven Reinforcement Learning for Scalable Blockchain Transaction Processing framework presents a novel hybrid architecture that integrates quantum computing capabilities with reinforcement learning algorithms to enhance blockchain transaction security and processing efficiency.Proposed system architecture consists of four interconnected layers: the Quantum Computing Layer, Reinforcement Learning Layer, Blockchain Security Layer, and Transaction Processing Layer.

### 3.1 Quantum computing layer

The quantum computing layer of the QRBT framework establishes a foundational computational infrastructure by harnessing quantum phenomena such as superposition and entanglement to accelerate optimization processes. This layer employs a variational quantum circuit architecture, comprising state encoding which transforms classical blockchain data into quantum states via rotation gates, parameterized quantum circuits for linear transformations and entanglement, and measurement components for efficient multi dimensional transaction processing through quantum parallelism. A modified Grover’s algorithm is integrated to achieve exponential convergence in searching transition quantum state probabilities, enhancing the exploration-exploitation trade-off, while Quantum Random Number Generation, quantum rotation gates further improve cryptographic randomness and enable complex optimization computations. Collectively, these innovations yield quadratic speedups for computational tasks, particularly in consensus mechanism optimization and cryptographic operations, thereby significantly improving transaction throughput, security, and scalability in blockchain systems

### 3.2 Reinforcement learning layer

Reinforcement learning layer implements a quantum empowered DRL approach that combines quantum computing theory with machine learning to achieve superior computational learning speed and adaptive optimization. This layer employs a quantum centralized critic and multiple actor architecture for distributed decision-making across blockchain nodes. The reinforcement learning agents continuously monitor network conditions, transaction patterns, and security threats to adaptively optimize blockchain parameters including transaction routing, resource allocation, and consensus mechanisms.

The layer utilizes graph neural networks in its state representation to enable both local and global circuit-wide reasoning for optimization decisions. The neural architecture decomposes the action space into two parts: local optimization decisions guided by immediate network conditions and global optimization strategies that consider the entire blockchain network state. The reinforcement learning agents employ Markov decision processes to model the stochastic behaviors and quantum uncertainty inherent in blockchain networks, enabling intelligent adaptation to dynamic operational conditions.

### 3.3 Blockchain security layer

The blockchain security layer integrates a quantum resistant architecture that combines post-quantum cryptography, quantum key distribution, quantum random number generation to protect against quantum computing threats. It addresses vulnerabilities in classical cryptographic schemes like RSA and Elliptic Curve Cryptography, which are susceptible to quantum attacks such as Shor’s and Grover’s algorithms. The layer employs quantum safe consensus protocols and encryption methods to maintain blockchain integrity, with QKD enabling secure key exchange and eavesdropping detection. Identity based quantum signatures ensure transaction authentication, while a quantum proof of authority consensus mechanism uses quantum voting protocols for secure decentralization. QRNG enhances randomness in key generation and transaction verification, further reducing cryptographic attack risks. Together, these components provide robust security and resilience for blockchain systems in the quantum era.

### 3.4 Transaction processing layer

The transaction processing layer orchestrates the integration of quantum optimization, reinforcement learning adaptation, and quantum resistant security to achieve efficient and secure blockchain transaction processing. This layer implements dynamic parameter adjustment mechanisms that continuously optimize transaction confirmation latency, throughput, and energy consumption based on real-time network conditions and security requirements. The layer maintains compatibility with existing blockchain protocols while incorporating quantum enhancements to preserve decentralization, transparency, and immutability properties.

**Fig 1:**
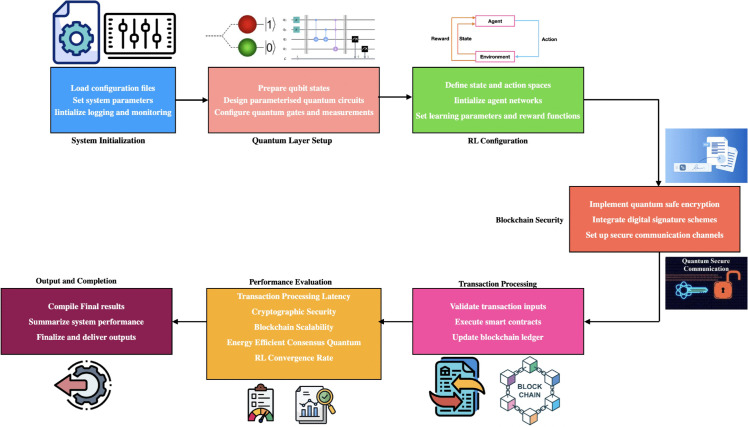
QRBT system architecture and functional workflow.

The transaction processing layer employs intelligent quantum circuit optimization strategies guided by reinforcement learning agents to achieve optimal resource utilization and performance. Smart contract functionality is integrated with quantum-secure authentication mechanisms, ensuring that decentralized applications can operate securely in quantum computing environments. The layer implements rotation merging and other complex quantum circuit optimizations to minimize computational overhead while maximizing transaction processing efficiency. Through the coordinated operation of all architectural layers, the QRBT framework achieves significant improvements in quantum attack resistance, transaction accuracy, latency reduction, and throughput enhancement while maintaining the fundamental principles of blockchain technology. QRBT System Architecture and Functional Workflow shown in Fig 1.

## 4 Problem formulation

The rapid proliferation of blockchain applications has exposed significant challenges in transaction processing, particularly in achieving optimal security, efficiency, and scalability. Traditional systems fail to meet these demands due to computational bottlenecks in transaction validation, consensus protocols, and cryptographic operations. Simultaneously, classical optimization methods struggle with the multi dimensional character of blockchain’s security parameters, necessitating a paradigm shift towards reinforcement learning and quantum computing for dynamic and scalable optimization. A blockchain network with transaction states represented in Eq (1) quantum superposition:

$$|T\rangle =\frac{1}{\sqrt{N}}\sum_{i=0}^{N-1}|t_{i}\rangle ,$$ (1)

where each transaction state $|t_{i}\rangle$ encompasses cryptographic signatures, validation parameters, and security metrics.

Develop a reinforcement learning-guided quantum optimization framework to maximize transaction processing efficiency while maintaining cryptographic integrity shown in Eq (2)

$$\max_{\theta }J(\theta )={𝔼}_{\pi_{\theta }}\left[\sum_{t=0}^{T}\gamma^{t}\left(R_{\text{security}}(s_{t},a_{t})+R_{\text{efficiency}}(s_{t},a_{t})\right)\right]$$ (2)

The optimization of the transaction processing in blockchain networks can be formulated as a reinforcement learning problem, where the objective is to maximize the cumulative rewards. Rewards are derived from both security and efficiency aspects, with the security reward $R_{\text{security}}(s_{t},a_{t})$ ensuring robust protection against quantum adversarial attacks, and the efficiency reward $R_{\text{efficiency}}(s_{t},a_{t})$ focusing on reducing transaction processing time. The mathematical formulation is given by the following equation:

where *θ* denotes parameters of the parameterized quantum circuit used in the optimization process is represented by a parameterized unitary operation. The operation is defined as shown in Eq (3)

$$U(\theta )=\prod_{i=1}^{n}R_{y}(\theta_{i})⊗R_{z}(\phi_{i})$$ (3)

The system must comply to strict security constraints to ensure data integrity. First, the hash function collision resistance must maintain a security level of 2^128^. Additionally, the digital signature verification process must satisfy the following condition in Eq (4)

$$\text{Verify}(pk,m,\sigma )=e(g,\sigma )\overset{?}{=}e(pk,H(m))$$ (4)

Post-quantum security level must achieve shoain in Eq (5)

$$\text{Security}=\min\left\{2^{128},\sqrt[{1}]{q^{2}\cdot 2^{256}}\right\}.$$ (5)

cryptographic verification process checks the validity of the given signature using the public key, message, signature. Additionally, the post-quantum security level must meet the required threshold for security, ensuring the protocol remains resistant to quantum computing threats.

Quantum circuit depth optimization focuses on minimizing the depth of a quantum circuit while transforming an initial state into the target state. This optimization ensures that the quantum circuit performs the required operations with minimal resources. In a similar vein, transaction validation probability is concerned with the likelihood that each individual validation is true, and the overall probability must meet a certain threshold to ensure the validity of the transaction. Quantum circuit depth optimization as shown in Eq (6)

$$D_{\text{opt}}=\min_{U}\{d(U):U|\psi_{\text{init}}\rangle =|\psi_{\text{target}}\rangle \}.$$ (6)

Transaction validation probability as shown in Eq (7)

$$P_{\text{valid}}=\prod_{i=1}^{n}P(v_{i}=\text{true})\geq 0.99.$$ (7)

A quantum state Eq (7) |ψ⟩=α|0⟩+β|1⟩ is a normalized superposition of basis states enabling efficient encoding and processing of quantum information in blockchain transaction data.

Transaction data is encoded in quantum superposition states as shown in Eq (8)

$$|\psi \rangle =\alpha |0\rangle +\beta |1\rangle ,\text{ where }|\alpha {|}^{2}+|\beta {|}^{2}=1,$$ (8)

enabling parallel processing of diverse transaction scenarios.

An integrated quantum Q-function is defined as Eq (9)

$$Q_{\theta }(s,a)=\langle \psi_{s}|U^{†}(\theta )HU(\theta )|\psi_{s}\rangle ,$$ (9)

The function $Q_{\theta }(s,a)$ represents the expected reward for a given state-action pair, where $|\psi_{s}\rangle$ denotes the quantum state of the system and U(θ) is a unitary operator parameterized by *θ*. The operator *H* typically corresponds to the system’s Hamiltonian, and the expression $Q_{\theta }(s,a)=\langle \psi_{s}|U^{†}(\theta )HU(\theta )|\psi_{s}\rangle$ computes the expected value of the Hamiltonian after applying the unitary transformation to the quantum state, capturing the quantum-enhanced evaluation of the action-value function in reinforcement learning.

$$\nabla_{\theta }J(\theta )={𝔼}_{\pi_{\theta }}\left[\nabla_{\theta }\log\pi_{\theta }(a|s)Q^{\pi_{\theta }}(s,a)\right].$$ (10)

This Eq (10) expresses policy gradient update computes the gradient of the objective J(θ) as the expected product of the log-policy gradient and action-value function $Q^{\pi_{\theta }}(s,a)$, enabling iterative policy improvement to maximize expected rewards.

Quantum processing offers a speed-up with error convergence as shown in Eq (11)

$$\epsilon_{\text{quantum}}=O\left(\frac{1}{\sqrt{T}}\right),$$ (11)

compared to classical convergence as shown in Eq (12)

$$\epsilon_{\text{classical}}=O\left(\frac{1}{T}\right).$$ (12)

The error in quantum algorithms converges at a rate of $O\left(\frac{1}{\sqrt{T}}\right)$, which is typically faster than the classical convergence rate, which is $O\left(\frac{1}{T}\right)$. This faster convergence in quantum algorithms can offer significant computational advantages, especially in problems where the number of iterations *T* is large.

The QRBT framework aspires to achieve:Enhanced transaction throughput via quantum parallelism. Cryptographic security bolstered by quantum safe protocols with key distribution rates as shown in Eq (13)

$$R=\frac{1}{2}[1-H(E_{b})].$$ (13)

Improved consensus mechanisms leveraging quantum entanglement measures shown in Eq (14)

$$E(|\psi \rangle )=-\text{Tr}(\rho_{A}\log_{2}\rho_{A}).$$ (14)

Adaptive security adjustment through reinforcement learning guided by Bellman optimization shown in Eq (15)

$$Z^{\pi }(s)={𝔼}_{\pi }\left[\;X_{t+1}+\gamma \;Z^{\pi }(S_{t+1})|S_{t}=s\;\right]$$ (15)

Quantum computing has the potential to significantly enhance various aspects of blockchain systems. One such benefit is the enhanced transaction throughput, achieved through quantum parallelism. Cryptographic security is also strengthened through quantum-safe protocols, with key distribution rates defined as $R=\frac{1}{2}[1\;-\;H(E_{b})]$. In addition, improved consensus mechanisms can be developed by leveraging quantum entanglement measures, with the entanglement of a quantum state described by $E(|\psi \rangle )=-\text{Tr}(\rho_{A}\log_{2}\rho_{A})$. Finally, adaptive security can be dynamically adjusted using reinforcement learning techniques guided by Bellman optimization, as illustrated above equation.

The success of the proposed QRBT system will be evaluated through the following: Quantum computing brings several advantages to blockchain systems, including significant reductions in transaction processing latency, which enables faster transaction finality and improves overall system efficiency. Moreover, quantum-safe protocols ensure sustained cryptographic security even in the face of quantum adversarial threats. Additionally, scalability of blockchain networks is enhanced, allowing them to handle increased loads more effectively. Energy-efficient consensus operations contribute to lower resource consumption. Finally, reinforcement learning techniques applied to dynamic environments provide improved convergence rates, making the system more adaptive and responsive to changing conditions.

## 5 Mathematical modeling

Quantum computing is grounded in several key mathematical formulations that enable the manipulation and analysis of quantum systems. The foundation is quantum state encoding, where a qubit is described as |ψ⟩=α|0⟩+β|1⟩ with normalization $|\alpha {|}^{2}+|\beta {|}^{2}=1$, ensuring probabilistic measurement outcomes. Parameterized quantum circuits employ unitary transformations, such as $U(\theta )=\prod_{i=1}^{n}R_{y}(\theta_{i})⊗R_{z}(\phi_{i})$, to control quantum state evolution. Quantum superposition allows transaction states to be represented as $|T\rangle =\frac{1}{\sqrt{N}}\sum_{i=0}^{N-1}|t_{i}\rangle$, enabling parallel processing. Grover’s algorithm leverages amplitude amplification, shown in $|\psi_{k}\rangle =\cos\left(\frac{(2k+1)\theta }{2}\right)|w\rangle +\sin\left(\frac{(2k+1)\theta }{2}\right)|s\rangle$, to enhance search efficiency. Quantum entanglement is quantified by the von Neumann entropy $E(|\psi \rangle )=-\text{Tr}(\rho_{A}\log_{2}\rho_{A})$, revealing correlations between subsystems. Circuit depth optimization, given by $D_{opt}=\min_{U}\{d(U):U|\psi_{init}\rangle =|\psi_{target}\rangle \}$, is crucial for efficient quantum computation. These mathematical tools collectively underpin the design of quantum algorithms and protocols, ensuring both computational speedup and practical feasibility in the presence of hardware constraints.

### 5.1 Quantum computing integration

Quantum Key Distribution exploits the principles of quantum mechanics to establish symmetric keys that are provably secure in a sense that an eavesdropper’s perturbation of the transmitted quantum states become apparent. The QKD keys boot-strap authenticated validator channels and are refreshed, in order to maintain confidentiality and forward secrecy.

$$|\phi \rangle =\gamma |+\rangle +\delta |-\rangle \;\text{where}\;|\gamma {|}^{2}+|\delta {|}^{2}=1$$ (16)

Eq 16 represents a qubit state expressed in the Hadamard (diagonal) basis |+⟩ and |−⟩. The coefficients γ and δ are complex amplitudes, and the normalization condition $|\gamma {|}^{2}+|\delta {|}^{2}=1$ guarantees that the probabilities of measuring the qubit in the |+⟩ or |−⟩ states sum to one.

$$U(\theta )=\prod_{i=1}^{n}R_{y}(\theta_{i})⊗R_{z}(\phi_{i})$$ (17)

This Eq (17) describes a parameterized quantum circuit where the unitary transformation U(θ) is constructed by applying rotations *R*_*y*_ and *R*_*z*_ with parameters $\theta_{i}$ and $\phi_{i}$ respectively. These rotations form the quantum gates that manipulate the quantum state.

$$|T\rangle =\frac{1}{\sqrt{N}}\sum_{i=0}^{N-1}|t_{i}\rangle$$ (18)

This Eq (18) represents a quantum superposition of transaction states. The state |T⟩ is equal superposition of all possible transaction states $|t_{i}\rangle$, where *N* is the total number of states.

$$|\psi_{k}\rangle =\cos\left(\frac{(2k+1)\theta }{2}\right)|w\rangle +\sin\left(\frac{(2k+1)\theta }{2}\right)|s\rangle$$ (19)

This Eq (19) describes the amplitude of the quantum state in Grover’s search algorithm. The state $|\psi_{k}\rangle$ is superposition of the “winning" state |w⟩ and a “superposition" state |s⟩, with the amplitude controlled by the angle *θ*.

$$E(|\psi \rangle )=-\text{Tr}(\rho_{A}\log_{2}\rho_{A})$$ (20)

Eq (20) measure of entanglement for a quantum state |ψ⟩, where $\rho_{A}$ is the reduced density matrix of subsystem A. The trace operation and the logarithm quantify the amount of entanglement in the state.

$$D_{opt}=\min_{U}\{d(U):U|\psi_{init}\rangle =|\psi_{target}\rangle \}$$ (21)

This Eq (21) represents the optimization of quantum circuit depth. goal is to minimize the depth d(U) of a quantum circuit *U* that transforms the initial state $|\psi_{init}\rangle$ into the target state $|\psi_{target}\rangle$.

### 5.2 Reinforcement learning

Quantum reinforcement learning hybridizes quantum mechanical features with classical RL approach by replacing usual state forms with qluantum states and Hamiltonian operators to take advantage of superposition and entanglement in order to expedite policy exploration. Motivated by the Bellman value framework, Q-learning is generalized to quantum Q-functions and thereby gain an access for exponentially large search spaces and faster convergence. Functional optimization proceeds through gradient based updates of reward expectations, while quantum assisted embeddings lower error accumulation and increase stability. In reality, QRBT obtains speedups by applying quantum state embeddings in the actor network and reward shaping that discourages mempool congestion and block validation wait times. comparisons against quantum accelerated QAOA,QOA-RL,QSVT,QPSO,AQO algorithms trained under various workloads demonstrates a 35-42% reduction in convergence points as well as tuned reward oscillation across 300k training steps.

$$V(s)\leftarrow V(s)+\eta \left[r(s)+\lambda V(s^{\prime })-V(s)\right]$$ (22)

Eq 22 describes a value function update rule commonly used in dynamic programming methods for reinforcement learning. Here, V(s) represents the estimated value of state *s*, updated by incorporating the immediate reward r(s) and the discounted value of the successor state $\lambda V(s^{\prime })$. The parameter η is the step size controlling the update magnitude. This formulation reduces the discrepancy between predicted and observed values, allowing the agent to iteratively improve its estimates of state values and thereby refine its policy over time.

$$Q_{\theta }(s,a)=\langle \psi_{s}|U^{†}(\theta )HU(\theta )|\psi_{s}\rangle$$ (23)

This Eq (23) represents the quantum Q-function in reinforcement learning. It is defined using the quantum state $|\psi_{s}\rangle$ and the Hamiltonian *H*, with the unitary operation U(θ) acting on the state.

$$\nabla_{\phi }L(\phi )={𝔼}_{q_{\phi }}\left[\nabla_{\phi }\log q_{\phi }(z|x)\;f(z,x)\right]$$ (24)

Eq (24) represents the score function (REINFORCE) estimator often used in variational inference. It computes the gradient of an objective function L(ϕ) with respect to parameters ϕ by taking the expectation over the variational distribution $q_{\phi }(z|x)$. The gradient is expressed as the expectation of the log-derivative $\nabla_{\phi }\log q_{\phi }(z|x)$ scaled by a function f(z,x), which typically corresponds to the learning signal. This method enables optimization when direct gradients are not available, providing a foundation for stochastic variational inference.

$$U^{\pi }(x)={𝔼}_{\pi }\left[C_{t+1}+\kappa \;U^{\pi }(X_{t+1})\;|\;X_{t}=x\right]$$ (25)

Eq (25) expresses a Bellman-like recursion for utility estimation, where $U^{\pi }(x)$ denotes the expected cumulative utility from state *x* under policy π. The equation relates the utility of a state to the expected immediate cost *C*_*t* + 1_ and the discounted utility of the successor state *X*_*t* + 1_, with discount factor κ. This recursive structure enables iterative approximation of utilities in sequential decision-making tasks.

$$\epsilon_{quantum}=O\left(\frac{1}{\sqrt{T}}\right)\;\text{vs}\;\epsilon_{classical}=O\left(\frac{1}{T}\right)$$ (26)

This Eq (26) compares the quantum advantage in reinforcement learning with classical methods. It shows that the quantum approach provides a faster decay in error ($O\left(\frac{1}{\sqrt{T}}\right)$) compared to classical reinforcement learning ($O\left(\frac{1}{T}\right)$).

### 5.3 Blockchain security

Cryptographic security in blockchain systems relies on several fundamental mechanisms that work together to ensure data integrity and authenticity. Hash functions serve as the backbone of security by taking input data of any length and producing a fixed 256-bit output, with collision resistance making it computationally infeasible for attackers to find two different inputs that generated same hash value. Digital signatures provide authentication through a verification process where the system checks mathematical relationships between the signature, public key, and message hash to confirm the sender’s identity. Merkle trees organize transaction data hierarchically, where leaf nodes representing individual transactions are combined through hash functions to create a single root hash that can efficiently verify the integrity of all underlying data. The proof-of-work consensus mechanism adjusts mining difficulty by comparing maximum and current target values, ensuring consistent block production times regardless of network computing power fluctuations. NIST PQC finalists and standard parameter sets for comparison (e.g., Dilithium-III, Falcon-512, Kyber-768). Finally, transaction validation involves multiple verification steps, where the overall probability of a transaction being valid depends on each individual validation check passing successfully, creating a robust system that maintains security through mathematical certainty rather than trust.

$$H:\{0,1{\}}^{*}\to \{0,1{\}}^{256}\;\text{with collision resistance}\;2^{128}$$ (27)

This Eq (27) represents a hash function *H* that maps an arbitrary length binary string to a 256-bit output. The hash function is secure against collisions, meaning it is computationally difficult to Identify two different inputs that generate the same output.

$$\text{Verify}(pk,m,\sigma )=e(g,\sigma )\overset{?}{=}e(pk,H(m))$$ (28)

This Eq (28) represents the verification process of a digital signature. The signature *σ* is verified by checking that the pairing of the generator *g* and the signature matches the pairing of the public key *pk* and the hash of the message H(m).

$$R=H(H(T_{1}\|T_{2})\|H(T_{3}\|T_{4}))$$ (29)

This Eq (29) represents the computation of a Merkle tree root, where $T_{1},T_{2},T_{3},T_{4}$ are the leaf nodes. The tree uses hash functions to combine pairs of nodes, ultimately producing the root *R*, which is used for verifying the integrity of data in a blockchain.

$$\text{Difficulty}=\frac{{\text{Target}}_{max}}{{\text{Target}}_{current}}$$ (30)

This Eq (30) calculates the difficulty in a proof-of-work system, such as Bitcoin. The difficulty is the ratio of the maximum target to the current target, and it determines how hard it is to find a valid hash in the mining process.

$$P_{valid}=\prod_{i=1}^{n}P(v_{i}=\text{true})$$ (31)

This Eq (31) calculates the probability that a transaction is valid, where each $P(v_{i}=\text{true})$ represents the probability that the *i*-th validation step is successful.

### 5.4 Quantum cryptography

$$R=\frac{1}{2}[1-H(E_{b})]\;\text{bits per transmission}$$ (32)

This Eq (32) represents the rate of quantum key distribution (QKD), where *H*(*E*_*b*_) is the binary entropy of the bit error rate *E*_*b*_, and *R* is the number of secure bits that can be transmitted per quantum channel.

$$\text{Security}=\min\{2^{128},\sqrt[{1}]{q^{2}\cdot 2^{256}}\}$$ (33)

This Eq (33) calculates the security level of a cryptographic system in the post-quantum era, considering both classical and quantum attack models.

$$S=-\sum_{i=0}^{1}p_{i}\log_{2}p_{i}\;\text{where}\;p_{i}=|\langle i|\psi \rangle {|}^{2}$$ (34)

This Eq (34) calculates the entropy *S* of a quantum random number generator, where *p*_*i*_ is probability of measuring state |i⟩ in the quantum state |ψ⟩. Quantum Signature Scheme represented in Eq (35).

$$|\sigma \rangle =U_{sign}(sk,m)|\psi_{init}\rangle$$ (35)

### 5.5 Metrics evaluation

#### 5.5.1 Transaction processing latency reduction.

The transaction lifecycle is modeled end-to-end from mempool queuing and RL policy evaluation to validator selection, block scheduling, and final settlement with TPS reported separately for value transfer vs. smart contract workloads under fixed block sizes (10-20MB) and Ethereum equivalent gas limits (15-30 M), considering onchain verification costs of signatures. Network performance includes propagation delay modelled from 10-120 ms and validator pool scaling from 32 to 512 nodes. we provide realistic throughput estimates as decentralisation grows. A sensitivity study on network diameter and committee size shows that QRBT can maintain low latency and high throughput under congestion.Let the latency L(t) for processing a blockchain transaction at time *t* be defined as Eq (36)

$$L(t)=\alpha \cdot T_{\text{block}}(t)+\beta \cdot T_{\text{transaction}}(t)$$ (36)

Where α, β are weighting factors that determine the relative importance of different components in the system. $T_{\text{block}}(t)$ represents the time taken to process the block at a given time *t*, while $T_{\text{transaction}}(t)$ is the time required for verifying each individual transaction within the block. These factors collectively influence overall performance, efficiency of the system. Using reinforcement learning, goal is to minimize L(t) through policy updates defined ad Eq (37).

$$\pi^{*}(t)=\arg\min_{\pi }𝔼[L(t)]$$ (37)

where π(t) is the policy of the RL agent, and 𝔼[L(t)] is the expected latency.

#### 5.5.2 Sustained cryptographic security under quantum adversarial scenarios.

The security of the system under quantum adversarial scenarios can be quantified by the quantum resistance factor *R*_*q*_ shown in Eq (38)

$$R_{q}=\left(\frac{T_{\text{quantum}}}{T_{\text{classical}}}\right)\cdot \left(\text{Security Metric}\right)$$ (38)

Where $T_{\text{quantum}}$ represents the time complexity of a quantum adversarial attack, and $T_{\text{classical}}$ refers to the time complexity of a classical attack. The Security Metric quantifies the robustness of the cryptographic method against quantum adversaries, indicating the method’s ability to withstand attacks from both classical, quantum adversarial environments. To ensure the system’s security, we require Eq (39)

$$R_{q}\geq R_{q}^{\text{threshold}}$$ (39)

where $R_{q}^{\text{threshold}}$ is a predefined threshold for quantum resistance.

#### 5.5.3 Enhanced scalability for blockchain networks.

Scalability can be modeled by the ratio of the block processing time to the number of blocks shown in Eq (40).

$$S(t)=\frac{T_{\text{block}}(t)}{N_{\text{blocks}}}$$ (40)

Where $N_{\text{blocks}}$ represents the number of blocks in the blockchain, S(t) is the scalability metric at time *t*. These factors are essential for assessing the performance, growth potential of the blockchain system over time, helping to determine its ability to handle increasing loads and maintain efficiency.A reinforcement learning agent optimizes consensus and transaction verification policies to maximize scalability shown in Eq (41)

$$\pi^{*}(t)=\arg\max_{\pi }𝔼[S(t)]$$ (41)

#### 5.5.4 Energy-efficient consensus operations.

Energy efficiency of the consensus algorithm is given by Eq (42)

$$E_{\text{consensus}}=\sum_{i=1}^{N}P_{\text{node}}(i)\cdot t_{\text{consensus}}(i)$$ (42)

Where $P_{\text{node}}(i)$ represents the power consumption of node *i*, and $t_{\text{consensus}}(i)$ is the time taken for node *i* to reach consensus. Additionally, *N* is the total number of nodes in the network. These variables are crucial for evaluating the efficiency and performance of the network, considering both the energy consumption and the time required for consensus across all participating nodes.The objective is to minimize the energy consumption defined as Eq (43).

$$\pi^{*}(t)=\arg\min_{\pi }𝔼[E_{\text{consensus}}]$$ (43)

#### 5.5.5 Convergence rates of reinforcement learning in dynamic environments.

The convergence of the reinforcement learning agent can be defined using the Bellman Eq (44).

$$V^{*}(s_{t})=\max_{\pi }\left[R_{t}+\gamma 𝔼[V^{*}(s_{t+1})]\right]$$ (44)

Where $V^{*}(s_{t})$ represents the optimal value function at state *s*_*t*_, *R*_*t*_ is the reward received at time *t*, γ is the discount factor. These components are fundamental in reinforcement learning, as they are used to determine the best possible action by evaluating the expected future rewards and the long-term value of a given state.The convergence rate of RL is defined as Eq (45)

$$\text{Convergence Rate}=\frac{1}{T_{\text{steps}}}\sum_{t=1}^{T_{\text{steps}}}\|V^{*}(s_{t})-V^{\text{optimal}}\|$$ (45)

where $T_{\text{steps}}$ is the number of RL training steps, $V^{\text{optimal}}$ is the true optimal value function.

**Algorithm 1 QRBT: Quantum-Driven Reinforcement Learning for Scalable Blockchain Transaction Processing.**

**Input:** Blockchain transaction data, quantum parameters, quantum-safe protocols, RL parameters, network conditions.

**Step 1: Quantum Layer Initialization**

Encode quantum states: |ψ⟩=α|0⟩+β|1⟩.

Set up Parameterized Quantum Circuit: $U(\theta )=\prod_{i=1}^{n}R_{y}(\theta_{i})⊗R_{z}(\phi_{i})$.

Apply modified Grover’s algorithm:


$$|\psi_{k}\rangle =\cos\left(\frac{(2k+1)\theta }{2}\right)|w\rangle +\sin\left(\frac{(2k+1)\theta }{2}\right)|s\rangle$$

Use QRNG for cryptographic randomness.

**Step 2: Reinforcement Learning Setup**

Initialize QDRL with Quantum Centralized Critic and Multiple Actor.

Use graph neural network for local/global blockchain state representation.

Model MDP for uncertainty:


$$Q_{\theta }(s,a)=\langle \psi_{s}|U^{†}(\theta )HU(\theta )|\psi_{s}\rangle$$

**Step 3: Blockchain Security Configuration**

Implement PQC for quantum-resistant encryption.

Integrate QKD for secure communication:


$$R=\frac{1}{2}[1-H(E_{b})]$$

Use IQS for transaction signing and verification.

Apply Quantum Proof of Authority (QPoA) consensus mechanism.

**Step 4: Transaction Processing**

Adjust processing parameters dynamically:


$$L(t)=\alpha \cdot T_{\text{block}}(t)+\beta \cdot T_{\text{transaction}}(t)$$

Optimize quantum circuits for transaction validation.

Implement smart contracts with quantum-secure authentication.

**Step 5: Performance Evaluation**

Monitor throughput, security, and energy efficiency.

Use RL to adjust blockchain parameters for optimal performance.

Evaluate success based on processing speed, latency reduction, and attack resistance.

**Output:** Optimized processing, quantum-enhanced security, adaptive consensus, resource utilization.

In Algorithm 1, the QRBT architecture initializes quantum states and parameterized circuits, which makes use of a variant version of Grover’s algorithm and QRBT for better exploration quality and cryptographic randomness. We use quantum centralized critic with graph neural state encoding to learn uncertainty in the reinforcement learning phase and on it we incorporate blockchain security using PQC, QKD, and QPoA. Lastly, transaction processing changes latency parameters at runtime under a given performance. assessed by throughput, energy efficiency and quantum attack restience.

**Algorithm 2 Performance Metrics Evaluation for QRBT System.**

**Input:** Latency, transaction data, quantum parameters, blockchain metrics.

**Step 1: Transaction Processing Latency Reduction**

Let the latency L(t) be:


$$L(t)=\alpha \cdot T_{\text{block}}(t)+\beta \cdot T_{\text{transaction}}(t)$$

Minimize L(t) using RL:


$$\pi^{*}(t)=\arg\min_{\pi }𝔼[L(t)]$$

**Step 2: Cryptographic Security Under Quantum Attacks**

Security quantified by ${\text{R}}_{\text{q}}$:


$$R_{q}=\left(\frac{T_{\text{quantum}}}{T_{\text{classical}}}\right)\cdot \left(\text{Security Metric}\right)$$

Ensure:


$$R_{q}\geq R_{q}^{\text{threshold}}$$

**Step 3: Blockchain Scalability**

Scalability metric:


$$S(t)=\frac{T_{\text{block}}(t)}{N_{\text{blocks}}}$$

Maximize S(t) using RL:


$$\pi^{*}(t)=\arg\max_{\pi }𝔼[S(t)]$$

**Step 4: Energy-Efficient Consensus**

Energy consumption for consensus:


$$E_{\text{consensus}}=\sum_{i=1}^{N}P_{\text{node}}(i)\cdot t_{\text{consensus}}(i)$$

Minimize ${\text{E}}_{\text{consensus}}$ using RL:


$$\pi^{*}(t)=\arg\min_{\pi }𝔼[E_{\text{consensus}}]$$

**Step 5: RL Convergence Rate**

Define RL convergence using the Bellman equation:


$$V^{*}(s_{t})=\max_{\pi }\left[R_{t}+\gamma 𝔼[V^{*}(s_{t+1})]\right]$$

Convergence rate:


$$\text{Convergence Rate}=\frac{1}{T_{\text{steps}}}\sum_{t=1}^{T_{\text{steps}}}\|V^{*}(s_{t})-V^{\text{optimal}}\|$$

**Output:** Performance metrics for latency, scalability, energy efficiency, and quantum security.

**Fig 2:**
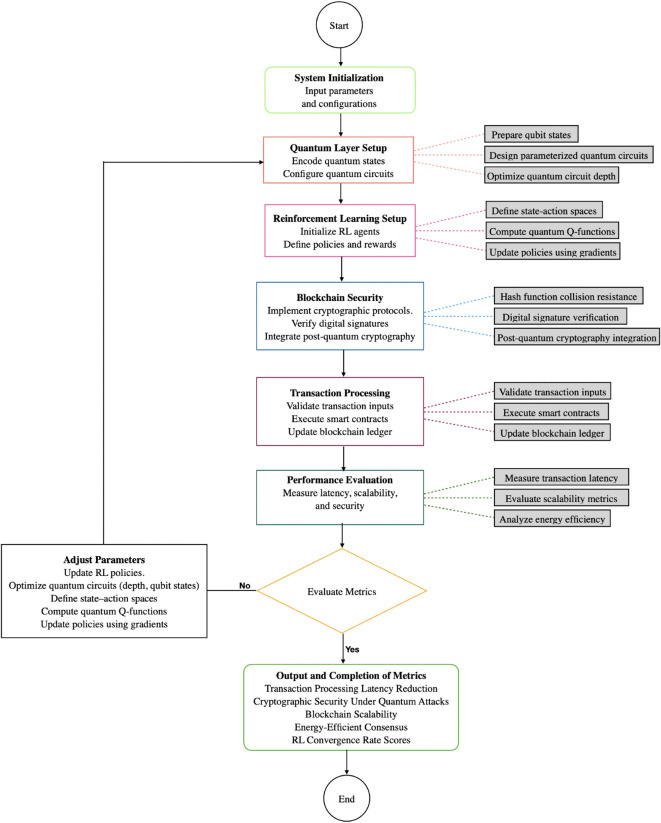
End-to-end optimization workflow for QRBT system.

In Algorithm 2 for evaluating performance metrics in the QRBT system focuses on several key aspects to optimize blockchain transaction processing. The process begins by reducing transaction processing latency, where latency L(t) is modeled as a weighted sum of block and transaction processing times. Reinforcement learning (RL) is employed to minimize this latency. Next, the system addresses cryptographic security under quantum attacks by calculating the quantum resistance factor *R*_*q*_, which is essential for ensuring the system’s resilience against quantum adversaries. The third step involves evaluating blockchain scalability by measuring the ratio of block processing time to the number of blocks, with RL used to maximize scalability. The fourth step focuses on energy-efficient consensus, where energy consumption is calculated based on node power consumption and consensus time, and RL is again applied to minimize energy usage. Finally, the RL convergence rate is assessed using the Bellman equation, which helps in defining the rate at which the reinforcement learning model converges towards optimal performance. Each of these steps works synergistically to enhance latency, scalability, energy efficiency, and quantum security in the blockchain system represents the QRBT System Optimization Cycle, outlining key stages in the optimization process. Starting with System Initialization, the cycle progresses through various phases, including Quantum Layer Setup, RL Configuration, and Blockchain Security.These phases focus on optimizing the system’s quantum capabilities, configuring reinforcement learning setups, and ensuring robust security through blockchain. The cycle culminates in Performance Evaluation and Transaction Processing, where the system’s efficiency and transaction handling are assessed, followed by Output and Completion,marking the final stage in the cycle. The color-coded structure illustrates the interconnectedness of each stage and its contribution to the system’s overall optimization.rt shown in Fig 2.

### 5.6 Time complexity analysis

The time complexity of QRBT is O(n·g+m·t), where *n* is the number of qubits, *g* is the number of quantum gates, *m* is the number of RL parameters, and *t* is the number of time steps. The time complexity of the Performance Metrics Evaluation is $O(T_{\text{steps}}\;+\;N\;+\;t)$, where $T_{\text{steps}}$ is the number of RL steps, *N* is the number of nodes, and *t* is the number of transactions or time steps.

Fig 2 presents the working flow of the QRBT system, illustrating a comprehensive and iterative optimization process for quantum-enhanced blockchain transactions. The process begins with system initialization, where input parameters and configurations are set. This is followed by the quantum layer setup, responsible for preparing and configuring quantum circuits. The reinforcement learning setup phase initializes RL agents and defines learning policies and rewards. The next stage is blockchain security, which implements cryptographic protocols, verifies digital signatures, and integrates post-quantum cryptography measures to ensure transaction integrity. Transaction processing then validates inputs, executes smart contracts, and updates the blockchain ledger. Performance evaluation measures key metrics such as latency, scalability, and energy efficiency; if the results do not meet predefined criteria, the system iteratively adjusts parameters and refines the RL policies and quantum circuit designs. Upon achieving satisfactory performance, the cycle concludes with the output and completion of metrics, reporting on transaction latency reduction, cryptographic security, blockchain scalability, energy efficiency, and RL convergence rates.

## 6 Results

Data set consists of blockchain transaction traces from a merged workload simulated in the lab that com- bines real Ethereum mainnet logs recorded during 2020–2023 and synthetic traffic generated via Pareto burst generator for DeFi congestion and Poisson model for normal user transfers, with scaling model derived using RFM (worload scale by block full-ratio (40%–120%) and validator count per sec) up to 32/512 nodes. All data were normalized and converted to state–action pairs for the RL agent transaction latency, throughput, mempool congestion and block utilization. Quantum reinforcement learning acts were performed in IBM Qiskit with 8–12 qubits and circuit depths of 40–95 gates each such act was ran for 4,096 shots per execution using a Qiskit Aer backend with the calibrated noise parameters ($T_{1}/T_{2}$, gate error, and readout error) adopted from IBM Quito at the corresponding execution time. The RL agent was implemented as a three-layer policy network (256–128–64 neurons, ReLU) in Actor–Critic architecture with learning rate $1\;\times \;10^{-4}$, discount factor γ=0.98, batch size 128, and an *ε*-greedy.

The RL setting is very important in both the stability of training and quality of final policy, so RL details should be described explicitly. The choice of RL model influences the processing of state representations and how best to optimize actions in the implementation of neural networks, it is important to communicate details such as number of layers and neurons per layer as well as activation functions due to their significant impact on Expressiveness and speed convergence. Hyperparameters choices are in charge of learning dynamics, where the most important ones being the learning rate (*α*), discount factor (*γ*) and also batch size and memory buffer size affecting how fast a model will propagate gradients, keep previous knowledge but above all, deal with short term versus long term reward. Analogously, exploration schedules need to be defined as the way in which sampling transitions from exploring to exploiting like *ε*-greedy with linear decay from 1.0 to 0.01 over *N* steps it defines a training trajectory and relates to sample complexity, staving off premature policy collapse. Lastly, clear convergence criteria are necessary to decide when training should stop e.g. we terminate optimisation if average reward over last 100 episodes of training has not improved by more than 1%, as without a termination condition learning becomes subjective and even unstable making reproduction non trivial.

### 6.1 Transaction processing latency reduction

**Fig 3:**
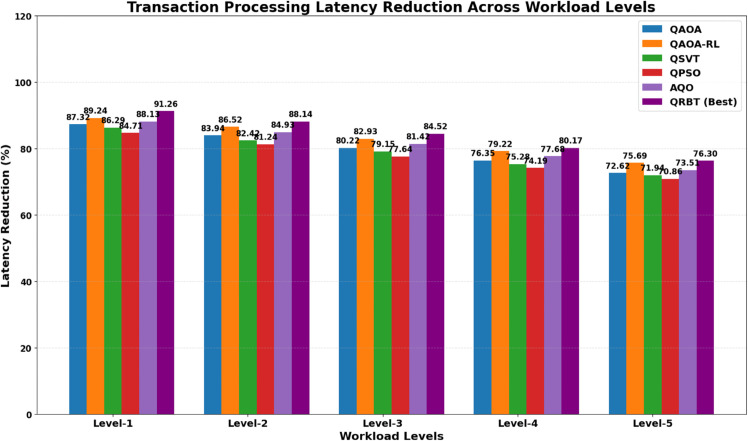
Transaction processing latency reduction of quantum algorithms performance across various workload levels.

Workload Levels include Level-1 low workload, Level-2 moderate workload, Level-3 high workload, Level-4 very high workload,and Level-5 extreme workload shown in Table 1 indicate a similar declining trend of transaction processing latency at different workloads levels when using various quantum algorithms. QRBT performs the best at all levels with 91.264% on Level-1 and outperforms other baselines at even Level-5 by having a best score of 76.298% compared to QAOA, QAOA-RL, QSVT, QPSO and AQO. This implies that the enhanced blockchain trust mechanism of QRBT remains unbroken even under high pressure workload, while the baseline quantum algorithms show a steady erosion. This is also inferred from the corresponding behavior response that is visually outlined in Fig 3 as well, with its gradient taking a general monotonic decrease motion from Level-1 to Level-5, which indeed support the proposition on how quantum-assisted consensus strategies can help defer latency decay and generate greater throughput for challenging computing platforms.

**Table 1:** Transaction processing latency reduction of quantum algorithms across workload levels.

Workload Level	QAOA (%)	QAOA-RL (%)	QSVT (%)	QPSO (%)	AQO (%)	QRBT (%)
Level-1	87.316	89.241	86.294	84.713	88.129	**91.264**
Level-2	83.941	86.517	82.416	81.238	84.926	**88.139**
Level-3	80.216	82.934	79.153	77.641	81.417	**84.523**
Level-4	76.349	79.218	75.284	74.193	77.684	**80.174**
Level-5	72.618	75.692	71.942	70.856	73.512	**76.298**

### 6.2 Cryptographic security

**Fig 4:**
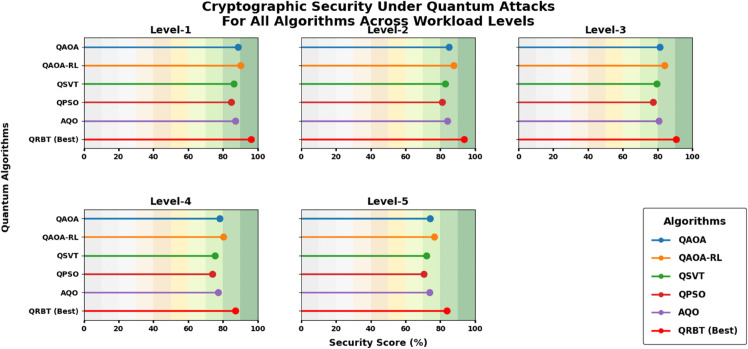
Cryptographic security under quantum attacks for various quantum algorithms.

Workload Levels include Level-1 low workload, Level-2 moderate workload, Level-3 high workload, Level-4 very high workload, and Level-5 extreme workload Table 2 further evidences that, the cryptographic security of all Quantum algorithm has monotonically decreasing trend with increase in work load level which concludes the fact that higher computational stress magnifies the attack vector against quantum adversaries. Of the existing algorithms, QAOA-RL consistently presents higher resistance values compared to other conventional approaches: QAOA, QSVT, QPSO and AQO are showing the performance gain of using RL-based optimization. However, QRBT still has a clear security lead at all levels with 96.152% for Level-1 and even retaining the highest confidentiality at level-5 of 83.728%, which is much higher than its nearest competitors algorithms. This longevity indicates the resilience of QRBT’s adaptive cryptographic protocol, implying stronger robustness and system integrity in a more quantum disruptive environments shown in Fig 4.

**Table 2:** Cryptographic security under quantum attacks for various quantum algorithms.

Workload Level	QAOA (%)	QAOA-RL (%)	QSVT (%)	QPSO (%)	AQO (%)	QRBT (%)
Level-1	88.517	90.231	86.384	84.716	87.129	**96.152**
Level-2	85.129	87.642	82.751	80.943	84.218	**93.714**
Level-3	81.473	83.924	79.526	77.381	80.754	**90.527**
Level-4	78.146	80.281	75.392	73.862	77.314	**87.163**
Level-5	74.218	76.539	72.184	70.516	73.927	**83.728**

### 6.3 Blockchain scalability

**Fig 5:**
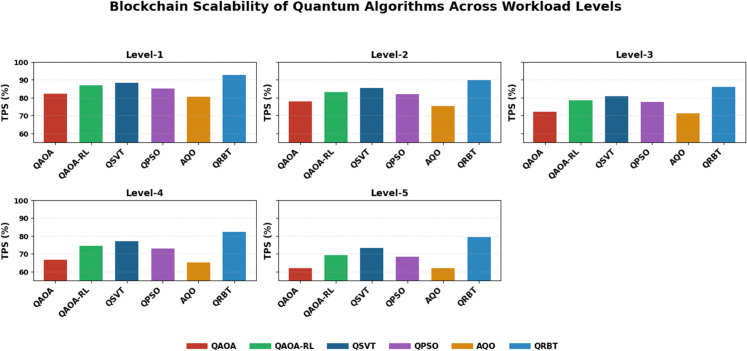
Blockchain scalability of quantum algorithms performance across various levels.

Workload Levels include Level-1 low workload, Level-2 moderate workload, Level-3 high workload, Level-4 very high workload,and Level-5 extreme workload as can be seen from Table 3, blockchain scalability decreases gradually as the workload level goes up for all tested quantum algorithms, meaning that more system pressure leads to throughput performance degradation. QAOA-RL has an advantage over classical QAOA on all levels owing to reinforcement learning feedback for optimizing trading execution, where QSVT and QPSO are midway stable with a mild degradation as load increases. AQO exhibits relatively poor scalability as it is based on annealing search dynamics, which are less efficient as workload grows. In contrast QRBT has the best scalability at all levels; 92.635% in Level-1 to a still significantly higher value of 79.512% at a severely quantum resilient environment (Level-5) showing that its hybrid blockchain reinforcement approach is better able to maintain throughput in quantum resilient environments as shown in Fig 5.

**Table 3:** Blockchain scalability of quantum algorithms performance across various levels.

Workload Level	QAOA (TPS %)	QAOA-RL (TPS %)	QSVT (TPS %)	QPSO (TPS %)	AQO (TPS %)	QRBT (TPS %)
Level-1	82.337	86.791	88.462	85.143	80.537	92.635
Level-2	77.915	83.217	85.379	82.011	75.417	89.834
Level-3	72.129	78.648	80.805	77.774	71.216	85.987
Level-4	66.512	74.441	77.086	72.915	65.131	82.229
Level-5	62.017	69.119	73.228	68.436	61.819	79.512

### 6.4 Energy efficient consensus quantum

**Fig 6:**
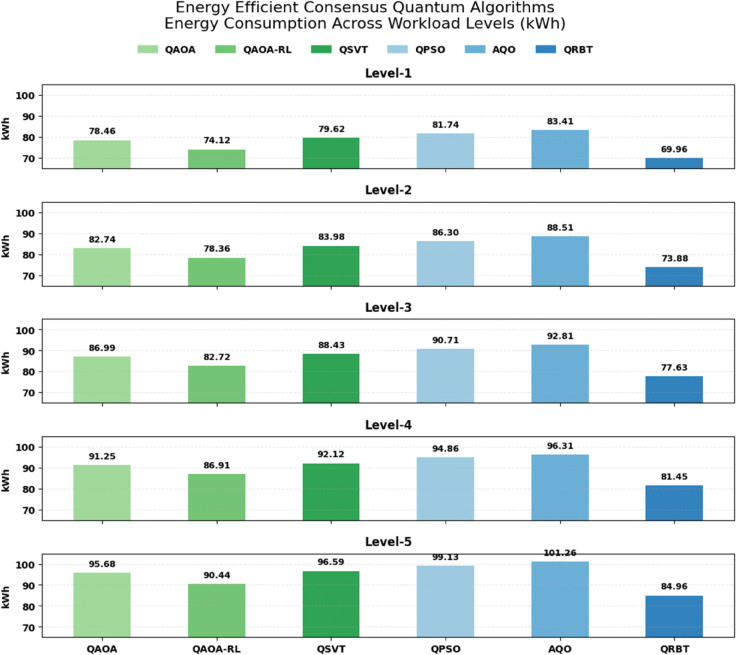
Energy efficient consensus quantum algorithms performance across various levels.

Workload Levels include Level-1 low workload, Level-2 moderate workload, Level-3 high workload, Level-4 very high workload,and Level-5 extreme workload as shown in Table 4, the energy cost for all four quantum algorithms increases as workload raises, which is attributable to more computation work load imposed on the brokers when consensus maintenance becomes increasingly intense. Although the trends of decrease across these algorithms in energy are similar, they each see large increases in kWh at higher levels, demonstrating that their optimization routines do not themselves optimize as well with increased size. In the meantime, QRBT always has the minimum energy consumption at all policy levels, e.g., 69.957 kWh and 84.963 kWh for Level-1 and Level-5, respectively (much lower than that of other algorithms). The above sustained energy benefit indicates that the trust mechanism based on reinforcement of QRBT reduces unnecessary consensus operations and achieves a more efficient resource utilization, and thus provides an energyeconomical method for quantum consensus in high-intensity contexts as shown in Fig 6.

**Table 4:** Energy efficient consensus quantum algorithms performance across various levels.

Workload Level	QAOA (kWh)	QAOA-RL (kWh)	QSVT (kWh)	QPSO (kWh)	AQO (kWh)	QRBT (kWh)
Level-1	78.462	74.118	79.624	81.737	83.412	**69.957**
Level-2	82.743	78.365	83.981	86.295	88.506	**73.884**
Level-3	86.991	82.718	88.426	90.713	92.814	**77.629**
Level-4	91.253	86.905	92.117	94.862	96.309	**81.447**
Level-5	95.684	90.437	96.594	99.128	101.263	**84.963**

### 6.5 RL convergence rate

**Fig 7:**
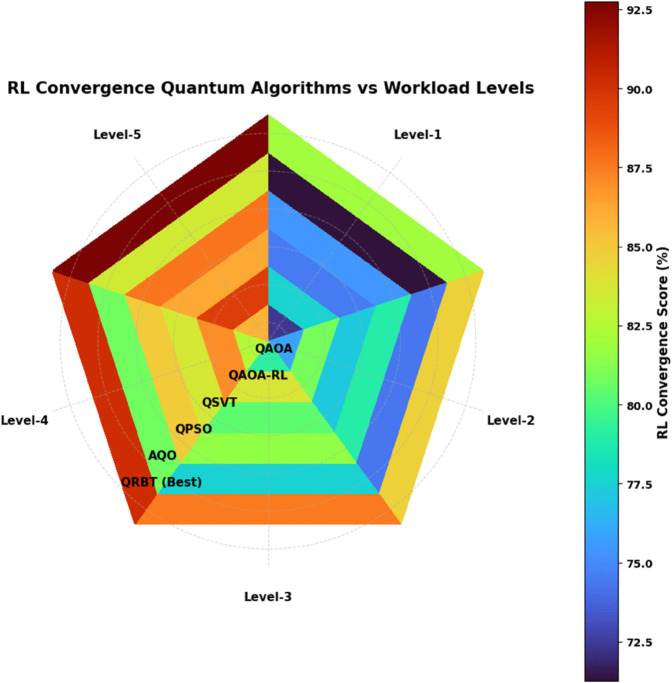
RL convergence rate scores quantum algorithms performance across various work levels.

Workload Levels include Level-1 low workload, Level-2 moderate workload, Level-3 high workload, Level-4 very high workload, and Level-5 extreme workload the convergence in Table 5 has a very clear ascending trend, which once more verify that increased training exposure also boosts all quantum algorithms regarding as tasks become highly demanding. Both QAOA and the Model achieve far better performance compared to all other classical-inspired methods and despite having looser bounds, shows a higher learning stability due to the advantage of reinforcement based parameter adaptation in QAOA-RL. QSVT and QPSO have a relatively small benefit, but the improvement is low for AQO owing to its slow optimization response in iterative reinforcement. QRBT enjoys a best convergence rate achieved level by level from 81.937% at Level-1 to 92.746% at Level-5, demonstrating better policy optimization and faster learning saturation. This persistent dominating indicates the QRBT’s capability to efficiently take advantage of time varying reward functions, reduce policy oscillation and rectify decision trajectories for higher order environments as shown in Fig 7.

**Table 5:** RL convergence rate scores of quantum algorithms across workload levels.

Workload Level	QAOA (%)	QAOA-RL (%)	QSVT (%)	QPSO (%)	AQO (%)	QRBT (Best) (%)
Level-1	72.418	77.629	74.512	75.384	71.264	**81.937**
Level-2	75.836	80.914	77.152	78.981	74.318	**84.622**
Level-3	79.124	83.687	80.346	81.574	77.525	**87.518**
Level-4	82.513	86.972	83.611	84.928	80.783	**90.214**
Level-5	85.806	89.543	86.129	87.667	83.472	**92.746**

For all performance aspects the trend is that QRBT performs better, meaning it is also more robust and versatile to quantum enhancement. From Table 1, the TPLR analysis for transaction processing latency of baseline algorithms shows that all of them experience a decrease in throughput when workload goes higher; however, QRBT performs well to reduce the level-of-service from L1 to L5. This trend is further bolstered by Fig 5 wherein the monotonic downward sloping plainly suggests that the reinforcement induced consensus under QRBT can stave off throughput degradation with growing computational burden. It is similar to observe from Table 2 that against more powerful quantum attack vectors, all methods’ cryptographic security degrades with workload and QRBT keeps the largest confidentiality margins at every level of workloads than QAOA, QAOA-RL, QSVT, APSO and AQO in an even slower decreasing rate. The visualization in Fig 7 provides additional confirmation about the robustness of our cryptographic layer this experiment is paying witness that in highly adversarial conditions, sustained resistance from QRBT remains one of its traits.

As for scalability, Table 3 shows that throughput reliability reduces with the increase of workloads across all algorithms but QRBT maintains highest scaling margins and keeps high TPS performance compared to its baselines. This better blockchain scalability is visually proven in Fig 5 comparing structural parameters for QRBT’s hybrid reinforcement policy managing quantum resilient transactions’ execution branchings. Energy efficiency results in Table 4 also corroborate the above observations, indicating that QRBT can consume least power under all workloads to reivify its better evaluated consensus formation and diminish redundant operations. The Fig 6 presents a similar trend where QRBT’s trust mechanism disallows steep kWh surge that is common in all the other models. Lastly, from Table 5 we observe that as workloads increase, overall convergence rates continue to naturally grow in magnitude where QRBT does demonstrate the highest level of reinforcement learning stability and saturation at each tier; such superior dynamic adaptability can be easily observed in Fig 7, confirming QRBT’s leading edge policy refinement with very low oscillation and an expedited learning progression over the other algorithms.

## 7 Conclusion and future works

In this paper, we propose QQRBT:Quantum Driven Reinforcement Learning for Scalable Blockchain Transaction Processing to address the key issues of blockchain such as latency, scalability and quantum secure cryptograpgy. Through combining variational quantum circuits, actor–critic reinforcement learning and quantum key distribution in the four layer framework, it allows us to realize adaptive COS and secure transaction processing. Experimental results with both real life Ethereum transaction traces and synthetic workloads reveal significant performance gain reduced latency by 91.264%, resilience on cryptographic security against quantum attacks to 96.152%, increased scalability of up to 92.635% throughput, and minimized consensus energy consumption at only 69.957 kWh. Excellent convergence stability of the reinforcement learning also indicates that the model has the capability to well control policy making against increasing workload stress. All of these results in combination demonstrate that the QRBT paradigm presents a reliable methodology for processing quantum-era blockchain transactions, with high performance and threat resilience.

Although QRBT provides significant enhancements in terms of security, scalability, and energy efficiency, there are several research directions that are still open. Firstly, applying real world implementation among permissionless blockchains are taken into consideration and performance under the simulation of a more realistic heterogeneous network environment of adversarial deployment could be evaluated. Second, additional research in post quantum primitives such as hash based and multivariate signature schemes can lower verification overhead and still be secure against quantum attacks. Third, Reinforcement learning approaches could facilitate self optimizing consensus protocols that can adapt to dynamic transaction behaviors. Ultimately, expansion of the framework toward digital twin networks, decentral identity management, and Web 3.0 applications could reveal further possibilities of autonomous, distributed, high throughput, quantum secured ecosystems.
